# cGMP-Prkg1 signaling PDE5 inhibition shelter cochlear hair cells and hearing function

**DOI:** 10.1186/2050-6511-14-S1-O27

**Published:** 2013-08-29

**Authors:** Marlies Knipper, Mirko Jaumann, Francois Paquet-Durand, Peter Ruth, Peter Sandner, Robert Feil, Jutta Engel, Lukas Rüttiger

**Affiliations:** 1 University of Tübingen, Department of Otolaryngology, Tübingen Hearing Research Centre (THRC), Molecular Physiology of Hearing, Tübingen, Germany; 2 University of Tübingen, Interfaculty Institute of Biochemistry, Tübingen, Germany; 3 University of Tübingen, Centre for Ophthalmology, Institute for Ophthalmic Research, Division of Experimental Ophthalmology, Tübingen, Germany; 4 University of Tübingen, Institute of Physiology II, Tübingen, Germany; 5 Department of Biophysics, Medical Faculty, Saarland University, Homburg, Germany; 6 University of Tübingen, Institute of Pharmacy, Department of Pharmacology and Toxicology, Tübingen, Germany; 7 Bayer HealthCare Pharmaceuticals, Global Drug Discovery - Common Mechanism Research, Pharma Research Centre Wuppertal, Wuppertal, Germany

## Background

Noise-induced hearing loss (NIHL) is a global health hazard with considerable pathophysiological and social consequences that has no effective treatment. In the heart, lung and other organs, cyclic guanosine monophosphate (cGMP) facilitates protective processes in response to traumatic events.

## Results

We therefore analyzed NIHL in mice with a genetic deletion of the gene encoding cGMP-dependent protein kinase type I (*Prkg1*) and found a greater vulnerability to and markedly less recovery from NIHL in these mice as compared to mice without the deletion. Prkg1 was expressed in the sensory cells and neurons of the inner ear of wild-type mice, and its expression partly overlapped with the expression profile of cGMP-hydrolyzing phosphodiesterase 5 (Pde5). Treatment of rats and wild-type mice with the Pde5 inhibitor vardenafil almost completely prevented NIHL and caused a Prkg1-dependent upregulation of poly (ADP-ribose) in hair cells and the spiral ganglion, suggesting an endogenous protective cGMP-Prkg1 signaling pathway that culminates in the activation of poly (ADP-ribose) polymerase.

## Conclusion

These data suggest vardenafil or related drugs as possible candidates for the treatment of NIHL.

